# Intentional partial odontectomy—a long-term follow-up study

**DOI:** 10.1186/s40902-017-0127-z

**Published:** 2017-10-05

**Authors:** Hyun-Suk Kim, Pil-Young Yun, Young-Kyun Kim

**Affiliations:** 1 Department of Oral and Maxillofacial Surgery, Section of Dentistry, Seoul National University Bundang Hospital, 300 Gumi-dong, Bundang-gu, Seongnam City, Gyunggi-do Korea; 2 Department of Dentistry and Dental Research Institute, School of Dentistry, Seoul National University, Daehak-ro 101, Jongno-gu, Seoul, 03080 Korea

**Keywords:** Hypercementosis, Mandibular nerve, Tooth ankyloses, Tooth extraction

## Abstract

**Background:**

The surgical extraction of the third molar is the most frequently encountered procedure in oral and maxillofacial surgery and is related with a variety of complications. This study examined the efficacy of intentional partial odontectomy (IPO) in the third molars which have no periapical lesions and are located near important anatomical structures such as inferior alveolar nerve.

**Methods:**

Seven patients (four males, three females, 39.1 ± 11.6 years), who received IPO to reduce the risk of inferior alveolar nerve injury (IANI), were followed long-term. The treated teeth were horizontally impacted third molars in the mandibular left (*n* = 5) or mandibular right (*n* = 4) areas and were all ankylosed with the surrounding alveolar bone. During the IPO, the bone around the crown was removed to expose the crown, and then the tooth was resected at cement-enamel junction (CEJ). Any secondary trauma to the healthy root was minimized and remained intact after primary suture.

**Results:**

The mean follow-up time was 63.2 ± 29.8 months, and all sites showed good bone healing after the crown removal. Also, sensory abnormality was not found in any patients after IPO. In one patient, the bone fragments erupted 4 months after IPO. In other patient, an implant placed on second molar site adjacent to the third molar that received IPO was explanted about 2 years after the patient’s persistent discomfort.

**Conclusions:**

In case where high risk of IANI exists, IPO may be chosen alternatively to surgical extraction to reduce the risk of nerve damage.

**Electronic supplementary material:**

The online version of this article (10.1186/s40902-017-0127-z) contains supplementary material, which is available to authorized users.

## Background

The impacted mandibular third molar which could result in pain or discomfort due to pericoronitis or dental caries needs to be extracted. Extracting the impacted mandibular third molar, however, is often challenging to dental surgeons because of related postoperative complications. The complications are highly related with the depth and position of impaction (i.e., mesio-angular, horizontal, vertical, and disto-angular), and the proximity to important anatomical structures such as the inferior alveolar nerve (IAN) canal [1]. Inferior alveolar nerve injury (IANI) is the most common complication during the extraction surgery [2].

In surgical extractions, the incidence of IAN damage varies from temporary paresthesia up to 8.1% and permanent discomfort up to 3.6% [3]. In cases of patients with a high risk of IANI, intentional partial odontecomy (IPO) could be considered as an alternative treatment option to complete extraction. IPO, which was first suggested by Knutsson et al. in 1989, is a procedure designed to reduce the risk of IANI by removal only the crown portion of the tooth, leaving the root in situ [4]. Pericoronitis, one of the reasons for extracting the mandibular third molar, is related with the persistence of the dental follicle that could pose as a source of infection. In such case, the removal of the crown portion with the follicles could subside inflammatory reaction occurred surrounding the tooth [3].

Despite the advantages that IPO could provide on an impacted tooth that is in a high risk of IANI, clinicians are reluctant to perform the procedure because residual roots could become a source of postoperative infection in the future. Therefore, IPO has been a controversial operation since it lacks a long-term follow-up of retained roots.

This retrospective study was designed to examine the fate and complications of residual roots through long-term follow-ups and to inform whether the IPO could be suggested as an alternative treatment for the impacted third molars.

## Methods

### Patients

In this study, seven patients (four males and three females; age 39.1 ± 11.6 years), who underwent IPO because of a high risk of intra- and postoperative complications, were followed long term. A total of nine mandibular third molars underwent IPO, five in the left and four in the right mandibles. The relation of the tooth to the ramus of the mandible, the relative depth of the third molar in the bone, and the position of the tooth in relation to the long axis of the second molar were radiographically evaluated following Pell & Gregory classification [5]. Based on this classification, the difficulty of extraction was evaluated using difficulty index described by Pederson [6]. Moreover, the preoperative status of the impacted tooth, such as ankylosis, hypercementosis, root shape, and IAN proximity, were recorded using radiographic views. Before the operation, all patients were fully given detailed accounts about the conventional extraction and IPO, and all consented to receive the latter procedure.

### Surgical procedure

**Fig. 1:**
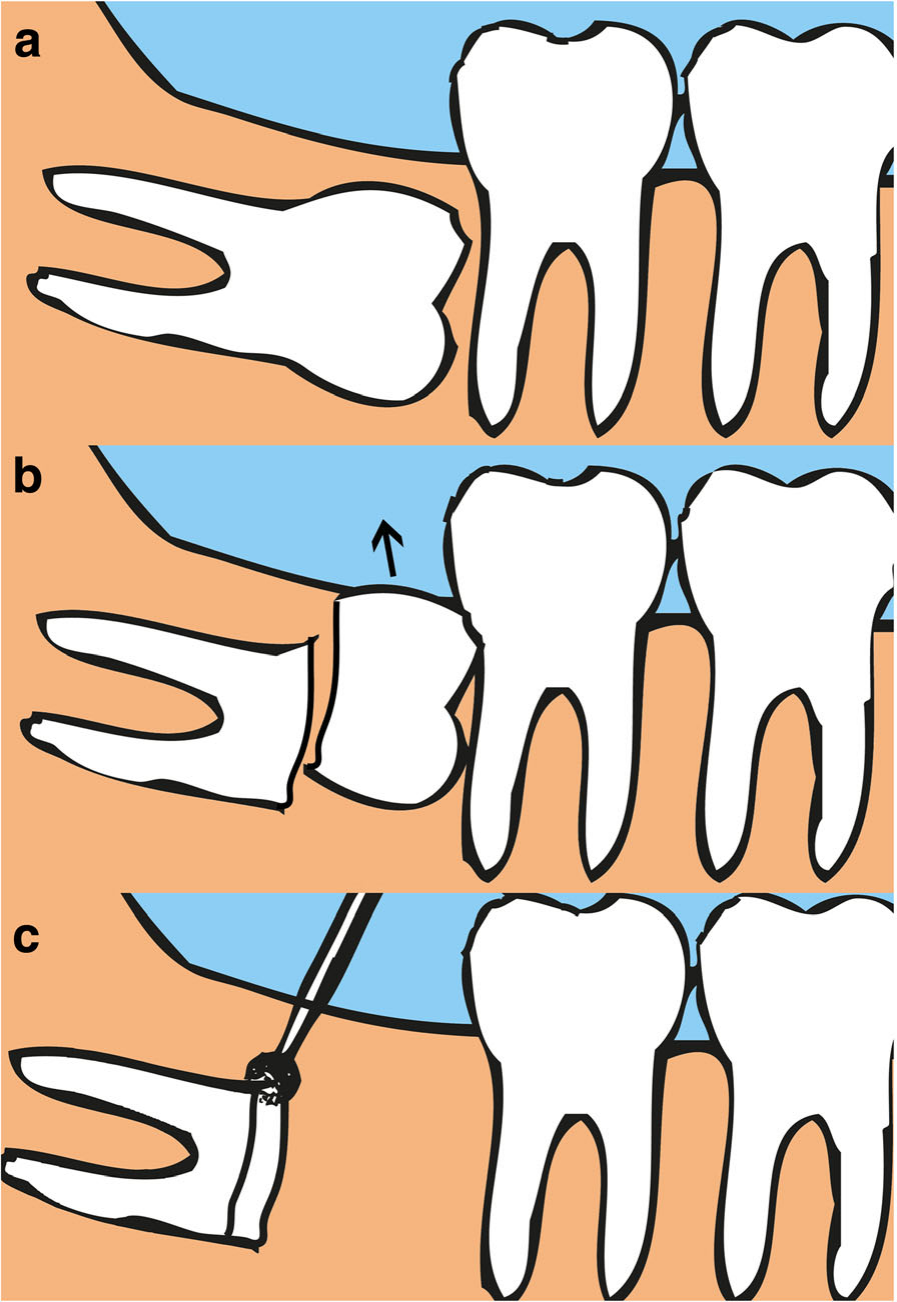
Intentional partial odontectomy. **a** Pre-operative mandibular third molar. **b** Coronectomy of the molar at cementoenamel junction (**c**). Trimming the cutting surface to 3 mm below the surrounding alveolar bone

All IPOs were performed by a highly experienced oral and maxillofacial surgeon. Under local anesthesia with 2% lidocaine including 1:100,000 epinephrine, a full-thickness mucoperiosteal flap was elevated with a periosteal elevator. Alveolar bones around the impacted crown were removed with a surgical bur to expose the cementoenamel junction (CEJ) of the tooth, and the tooth was sectioned at the junction. After decrowning, the cutting margin was trimmed to 3 mm below the adjacent bone level (Fig. 1). The mobility of the remaining root was checked, and any secondary trauma to the healthy roots was minimized. The surgical area was irrigated with normal saline and then sutured primarily with 3-0 black silk (Ethicon Sutures, Ltd., USA). Antibiotics (amoxicillin/clavulanate; Augmentin®, Ilsung Pharmaceuticals Co., Seoul, Korea) and a non-steroidal anti-inflammatory drug (talniflumate; Somalgen®, Kunwha Pharmaceutical Co., Seoul, Korea), and 100 mL of 0.1% chlorhexidine mouth gargling (Hexamedine®, Bukwang Pharm, Ansan, Korea) were prescribed for oral hygiene maintenance. Sutures were stitched out 1 week after the surgery. Any complications related with sensory abnormality, pain, infection, migration of remained roots, and influence to the adjacent tooth was evaluated clinically and radiographically.

## Results

The mean follow-up period was 63.2 ± 29.8 months, and all operation sites were evaluated clinically and radiographically. According to Pell & Gregory classification, there were six IIIC, two IIIB, and one IIC. Out of the nine teeth, four were mesioangular, three horizontal impactions, and two vertical impactions. Applying Pederson’s difficulty index, only one out of the nine cases was presumably “moderately difficult,” while the rest were “very difficult” extractions. Considering that the difficulty index does not take ankylosis/hypercementosis into account, actual levels of extractions presented in this article were very difficult even for a highly experienced oral surgeon (Table 1).

**Table 1:** Description of impacted third molars

#	Age	Sex	Site	Pell & Gregory classification		Pederson difficulty index	Pre-operative status				F/U (mo.)	Complication
							AK	HC	RS	IP		
1	48	F	#48	IIIC	M	7	+	−	C	+	118.4	trismus
2	59	M	#38	IIIC	H	8	+	+	C	+	91.5	–
3	39	M	#48	IIIC	M	7	+	−	C	+	65.8	–
4	32	F	#38	IIC	M	6	+	−	C	+	47.5	Bone fragment
5	32	F	#48	IIIC	V	9	+	−	C	+	46.8	–
6	43	F	#38	IIIC	M	7	+	−	D	+	32.7	pain, trismus
7	49	M	#38	IIIB	V	8	+	+	D	+	33.4	–
8	25	M	#48	IIIB	H	7	+	−	D	+	60.2	–
9	25	M	#38	IIIC	H	8	+	−	C	+	59.7	–

Difficulty index 3–4(slightly difficult), 5–6(moderately difficult), 7–10(very difficult)

*AK* ankylosis, *HC* hyper-cementosis, *RS* root shape, *IP* IAN proximity (≤ 2 mm), *M* mesio-angular, *H* horizontal, *V* vertical, *C* convergent, *D* divergent

According to the panoramic views, all molars were impacted close to the white line of IAN canal (≤ 2 mm), and all roots lost lamina dura suggesting that the teeth are ankylosed with the surrounding alveolar bone. Also, two teeth showed signs of hypercementosis, and root shape of the three teeth was divergent.

**Fig. 2:**
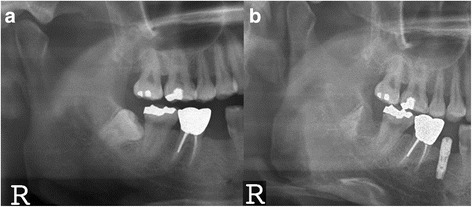
A panoramic view of pre- and post-IPO of the mandibular right third molar. **a** Pre-IPO. **b** 70 months after IPO

On the recall check, none of the patients had sensory abnormality such as paresthesia and hypoesthesia related with the surgical sites. Moreover, all the operation sites were covered with the healthy gingiva without postoperative infection signs. Radiographically, none of the residual roots that were surrounded by the intact bones migrated (Fig.2).

In one patient, a bony fragment erupted 4 months after IPO. The patient did not show any clinical symptoms related to it, and the fragment was simply removed and it did not cause the failure of IPO.

In another patient, an implant placed on mandibular second molar area adjacent to the third molar that received IPO was explanted about 2 years after because of persistent discomfort. However, in this case, the bone around the resected root was intact, and there was not a sign of infection in the IPO site. Therefore, the reason for implant failure is considered as the peri-implantitis.

One out of nine cases reported severe pain (Visual Analog Scale of 6 out of 10) a day after IPO, which alleviated after a week.

## Discussion

To avoid complications when a mandibular third molar is impacted close to an IAN canal, IPO is considered as an alternative treatment to the surgical extraction [6]. A study about IANIs after the conventional extraction and IPO reported that the incidence of the damage to the IAN was higher in complete extraction (19% of 102 cases) compared with IPO (3% of 94 cases) [7]. Another case-control study also concluded that IANIs as a result of IPO was not reported at all, whereas the conventional surgical extraction resulted to 5% IANIs [8]. Despite these positive reports on IPO, many dental surgeons are still reluctant to remain the root portions and worry about the postoperative complications because of a lack of studies reporting the long-term follow-up results [9].

As of disadvantage of IPO, migration of residual roots could happen. A study mentioned that 30% of remnant roots migrated towards the superior border of the mandible in the first year, and additional surgery for removal of roots was required [10]. Another study reported 6% incidence of later root removal after the remained root migrated far from the IAN canal [11]. In other hand, Dolanmaz stated that none of the 43 patients who were treated by IPO surgery required additional removal resulting from the subsequent root migration [12]. In our study (*n* = 9), there was no radiographic evidence of migration of the residual roots. It seems that hypercementosised and ankylosed roots covered with surrounding bone are not easily migrated from its original location.

IPO sometimes is known to cause an infection as one of the postoperative complications. IPO without complete removal of the dental follicle could lead to infection up to 5% [4]. Renton also reported 10–12%, relatively high incidence, of infection on operation sites after IPO [7]. Whereas, Dolonmaz showed that none of the 43 cases were related with postoperative infection, and Porgrel reported only one case of postoperative infection out of 50 IPO cases [13, 14]. In our study, postoperative infection was not observed in the IPO site. After the long periods of healing, the operation sites were covered with healthy gingiva. In one case (case #2), an implant placed adjacent to the third molar that received IPO failed 2 years after implantation. In this case, the bone healing around the residual roots was normal, and the third molar area did not display any specific signs of abnormality upon visual exam and palpation tests. Therefore, in this case, the reason for implant failure is not resulted by IPO, but possibly by peri-implantitis.

Another concern of performing IPO is postoperative pain. Hatano Y et al. had reported high incidence of postoperative pain in the coronectomy group compared to the conventional extraction group, which had diminished within 1 week [9]. Hatano Y et al. proposed that possible reasons to the acute pain are a tight primary closure which could have caused high pressure inside the wound and a temporary pulpitis of the resected root. According to O’Riordan study [3], the resected pulp could result in hyperemia or inflammatory edema which could develop into pulp pathogenesis. Therefore, adequate irrigation and avoidance of any manipulation of vital pulp that could facilitate dentinal bridge around pulp chamber are important. In our study, one patient complained of severe pain (VAS 6) a day after IPO. The pain was relieved within 1 week. Although histopathologic section was not performed on the symptomatic IPO tooth, our assumption is that the pulp had temporary pulpitis which could have been caused by heat arose from inadequate coolant during the coronectomy.

In terms of risk of IANI, it is obvious that decrowning the highly risky, impacted mandibular third molar is safer than the complete extraction. Therefore, when dental surgeons encounter the third molar extraction cases, they should initially evaluate difficulties of extraction with radiographic findings. If the mandibular third molar is located too near an IAN canal, and if the roots have hypercementosis or ankylosis, a surgeon could consider IPO as a primary treatment option. However, patients should be sufficiently informed of the advantages/disadvantages of the IPO surgery and understand why this technique is necessary before the procedure starts. In regard to IPO, a long-term follow-up is important to evaluate patient’s discomfort including neuropathy, postoperative infection, and development of any pathology.

In our study, the mean follow-up period was 61.7 ± 27.8 months, which was enough to assess any complication of IPO. However, the sample size of this study is relatively small as compared with other studies reporting the results of IPO. Moreover, our study has no control group to compare the effectiveness of conventional extraction with IPO. Therefore, it is suggested that a long-term study with more cases should be conducted to evaluate the benefits of IPO and to compare with the traditional extraction method.

## Conclusions

In case where high risk of IANI exists, IPO may be chosen alternatively to surgical extraction to reduce the risk of nerve damage, but a larger sample size of similar follow-up period should be supplemented.

## Additional file

Additional file 1: Case form and result of data. (XLSX 14 kb)

## Abbreviations

CEJ: Cementoenamel junction
IAN: Inferior alveolar nerve
IANI: Inferior alveolar nerve injury
IPO: Intentional partial odontectomy
